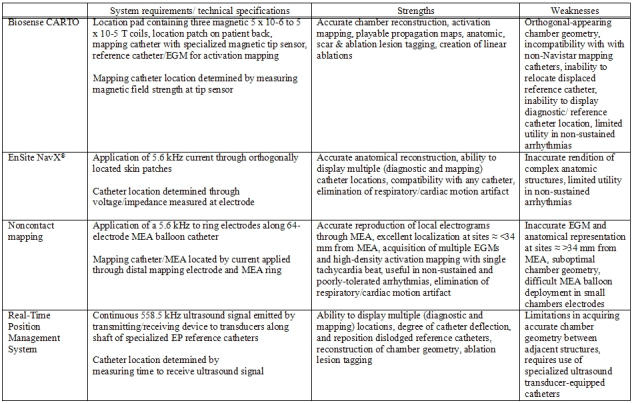# Principles of Electroanatomic Mapping

**Published:** 2008-02-01

**Authors:** Deepak Bhakta, John M Miller

**Affiliations:** Krannert Institute of Cardiology, Indiana University School of Medicine

**Keywords:** Ablation, arrhythmia, electrophysiologic testing, mapping system, electroanatomic mapping

## Abstract

Electrophysiologic testing and radiofrequency ablation have evolved as curative measures for a variety of rhythm disturbances.  As experience in this field has grown, ablation is progressively being used to address more complex rhythm disturbances.  Paralleling this trend are technological advancements to facilitate these efforts, including electroanatomic mapping (EAM).  At present, several different EAM systems utilizing various technologies are available to facilitate  mapping and ablation.  Use of these systems has been shown to reduce fluoroscopic exposure and radiation dose, with less significant effects on procedural duration and success rates.  Among the data provided by EAM are chamber reconstruction, tagging of important anatomic landmarks and ablation lesions, display of diagnostic and mapping catheters without using fluoroscopy, activation mapping, and voltage (or scar) mapping.  Several EAM systems have specialized features, such as enhanced ability to map non-sustained or hemodynamically unstable arrhythmias, ability to display diagnostic as well as mapping catheter positions, and wide compatibility with a variety of catheters.  Each EAM system has its strengths and weaknesses, and the system chosen must depend upon what data is required for procedural success (activation mapping, substrate mapping, cardiac geometry), the anticipated arrhythmia, the compatibility of the system with adjunctive tools (i.e. diagnostic and ablation catheters), and the operator's familiarity with the selected system.  While EAM can offer significant assistance during an EP procedure, their incorrect or inappropriate application can substantially hamper mapping efforts and procedural success, and should not replace careful interpretation of data and strict adherence to electrophysiologic principles.

Electrophysiologic (EP) testing and radiofrequency ablation (RFA) have evolved as curative measures for a variety of rhythm disturbances [1-3].  While originally applied towards relatively straightforward arrhythmias with a single discrete target site (such as atrioventricular [AV] nodal reentry or tachycardias associated with Wolff-Parkinson-White syndrome), they are increasingly being used to address more complex arrhythmias, including atypical atrial flutter, atrial fibrillation, and ventricular tachycardia.  This latter group of rhythm disturbances is often associated with significant underlying structural cardiac abnormalities, such as congenital, ischemic and post-surgical heart disease. Fortunately, the broader use of EP testing and RFA in such individuals has been accompanied by technological enhancements to address this progression. Electroanatomic mapping (EAM) is one such advancement, allowing operators to record intracardiac electrical activation in relation to anatomic location in a cardiac chamber of interest, during arrhythmia mapping.  Several EAM systems are currently available that accomplish these tasks. When applied properly, such technology allows one to accurately determine the location of arrhythmia origin, define cardiac chamber geometry in three dimensions, delineate areas of anatomic interest, and allow catheter manipulation and positioning without fluoroscopic guidance. These systems often simplify mapping efforts and can enhance procedural success, particularly in cases in which complex arrhythmias and unusual cardiac anatomy are encountered.

## Why use an EAM system?

While having an EAM system available for every procedure may seem like an advantage, their expense and the additional preparatory time must be justified.  This may be relatively easy to do when complex arrhythmias or cardiac anatomy are anticipated.  When available information suggests an arrhythmia mechanism located near important structures such as a manifest bypass tract originating near the AV node, EAM may allow for marking of such structures so that they may be avoided.  Similarly, when complex anatomy and areas of scar contributing to macroreentry are anticipated, EAM may allow demarcation of such regions so that they may be isolated.  Non-sustained tachycardias or rapid rhythm disturbances resulting in hemodynamic compromise lend themselves well to certain EAM systems, as suspected sites of tachycardia origin may be more accurately recorded and targeted for ablation than with fluoroscopy alone.  Chief among these, however, is EAM's ability to reliably allow catheter positioning without the use of fluoroscopy, a distinct advantage for both patient and operator.

Several studies have confirmed these advantages, with comparable success rates to conventional, non-EAM guided approaches. Fluoroscopy time and radiation dose are indeed reduced with EAM when compared to conventional mapping strategies.  This is true for a variety of rhythm disturbances including AV node reentry and bypass tract-mediated arrhythmias [4-7].  Variable results have been shown when EAM systems are applied to atrial flutter though overall reductions in these parameters have also been demonstrated [4,7-11]. Shortened procedure duration is also apparent in several of these series, though many have shown only a neutral effect of EAM on procedural time.

Perhaps the greatest impact of EAM is its application to facilitate pulmonary vein isolation for treatment for atrial fibrillation - EAM use is consistently associated with reduced fluoroscopy time, radiation dose, and procedure time [12-14]. However, these relative benefits of EAM systems must be weighed against their added expense, which was increased by 50% in one series [7].

## Biosense CARTO® mapping system

As the CARTO mapping system is arguably the most widely used mapping system, it will be the focus of the following discussion.

**Figure 1:**
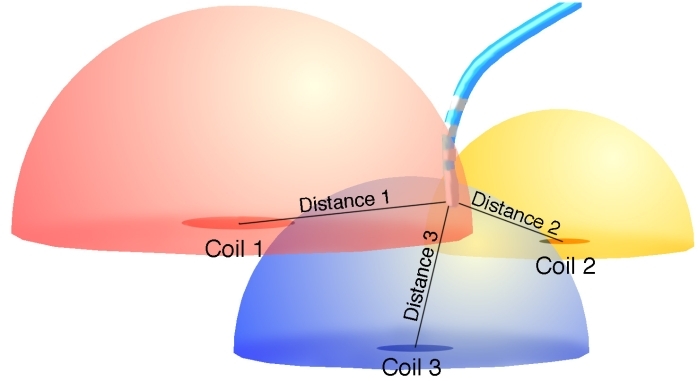
Illustration demonstrating operation of the Biosense CARTO electroanatomic mapping system.  Three separate coils emit a low-level magnetic field, diagrammatically represented by color-coded hemispheres.  The field strength from each coil is measured by a sensor within the tip of a specialized mapping/ablation catheter, and its position relative to each coil is then triangulated.

The CARTO mapping system (Biosense, Diamond Bar, CA, USA) utilizes a low-level magnetic field (5 x 10^-6^ to 5 x 10^-5^ Tesla) delivered from three separate coils in a locator pad beneath the patient. (Figure 1)  The magnetic field strength from each coil is detected by a location sensor embedded proximal to the tip of a specialized mapping catheter.  The strength of each coil's magnetic field measured by the location sensor is inversely proportional to the distance between the sensor and coil.  Hence, by integrating each coil's field strength and converting this measurement into a distance, the location sensor (and therefore, catheter tip location) can be triangulated in space [15]. The mapping catheter has proximal and distal electrode pairs, and a tip electrode capable of radiofrequency energy delivery. This catheter can be moved along a chamber's surface to record local endocardial activation times for arrhythmia mapping, while simultaneously recording location points to generate 3D chamber geometry.  Validation studies have shown CARTO to have substantial accuracy in navigating to single points, in returning to prior ablation sites, and in creating a desired length of ablation line [15-17].  Electrograms recorded from the specialized mapping (NaviSTAR) catheter showed excellent correlation with recordings from standard EP catheters [15].  Human validation studies have shown a similar level of spatial precision and accuracy, and realistic reconstruction of chamber geometry and electroanatomic activation during arrhythmia mapping [18].  Advantages of CARTO include accurate representation of chamber geometry and the capability of generating isochronal activation maps and playable propagation maps.  It also has the capability to record locations of important anatomic landmarks (such as the bundle of His), areas of electrical scar (to create voltage/scar maps) and vessels (coronary sinus [CS], pulmonary veins [PVs]).  CARTO also allows recording ablation lesion location facilitating creation of ablation lines.  Disadvantages include requiring the use of a specialized (NaviSTAR) catheter, inability to easily relocate a displaced reference catheter, somewhat orthogonal appearance to chamber geometry (as when a small number of location points are stored), and the inability to record or display the location of diagnostic/reference catheters.

## Set-up of the CARTO mapping system

### Location reference

Prior to arrhythmia mapping, a stable location reference must be established. This is accomplished by placing the location magnet, a triangular apparatus containing three magnetic coils, beneath the patient and table. The location of this magnet must be aligned anywhere inside a defined circumference at the start of the procedure.  A reference patch is affixed to the patient's back roughly overlying the cardiac chamber of interest. Should the location reference magnet or patch become displaced during the procedure, their original location is recorded by CARTO to allow proper repositioning. This allows for accurate tracking of mapping catheter position, consistency of anatomic landmark and ablation lesion locations, and precise reconstruction of chamber geometry.

### Activation mapping with CARTO

**Figure 2:**
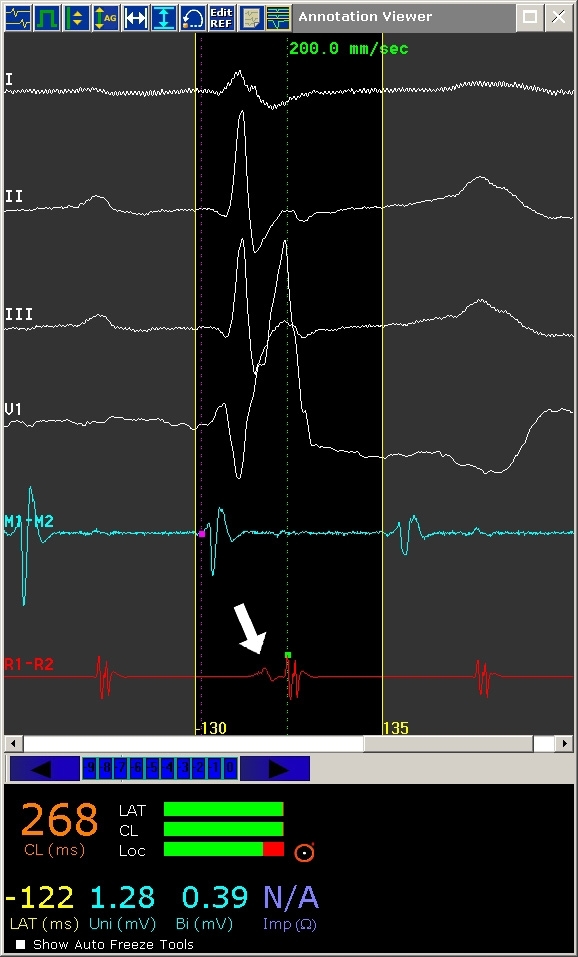
Annotation window for the CARTO mapping system.  Represented from top to bottom are ECG leads I, II, III, and V1, followed by distal mapping catheter electrogram (M1-M2) and reference electrogram (R1-R2).  In this example, atrial flutter (cycle length 268 ms) is being mapped and a mid-CS recording site has been selected as the reference channel.  The clear area surrounded by fine yellow lines is the timing window of interest; the left and right boundaries representing operator-defined earliest (-130 ms) and latest (+135 ms) endocardial activation, relative to the reference electrogram.  The magenta dot in the mapping catheter electrogram indicates the timing onset of local activation while the green dot (arrow) indicates the timing onset of the reference electrogram.

Once the location reference has been stably situated, an appropriate timing reference and window must be selected. (Figure 2)  The timing reference is any arbitrarily selected recording (intracardiac electrogram [EGM] or surface ECG lead) representative of activation of the chamber of arrhythmia origin (i.e. proximal CS atrial EGM chosen for mapping atrial tachycardia [AT]). Intracardiac EGMs are often selected as the timing reference as these are generally more consistent in appearance and precise in timing than surface ECG recordings, and consequently, are more reliable.  For rhythm disturbances where atrial activation is to be mapped (premature atrial complexes, AT, concealed bypass tract), the CS is a suitable choice as a timing reference, due to its stable positioning and lower likelihood of being dislodged and disrupting activation mapping (compared to a high right atrial catheter, for example, that may be dislodged by a roving mapping catheter). Any component of the reference electrogram may be chosen for a timing reference, including maximum (peak positive) deflection, minimum (peak negative) deflection, maximum upslope (dV/dT) or maximum downslope. The proximal/mid CS electrodes are often selected as a timing reference as the atrial EGM in these poles are often of greater amplitude and fidelity than their ventricular counterparts, preventing inappropriate timing using the ventricular EGM.  Such inaccurate results can be observed if the distal CS electrodes are selected as the timing reference, for example, where the ventricular EGM amplitude is equivalent to, or even larger than the atrial EGM. The ventricular EGM may then be inappropriately taken as the timing reference yielding an incorrect and confusing activation map. When mapping ventricular tachycardias or premature ventricular complexes, a right ventricular apical recording may be chosen as a stable timing reference for ventricular activation.

**Figure 3:**
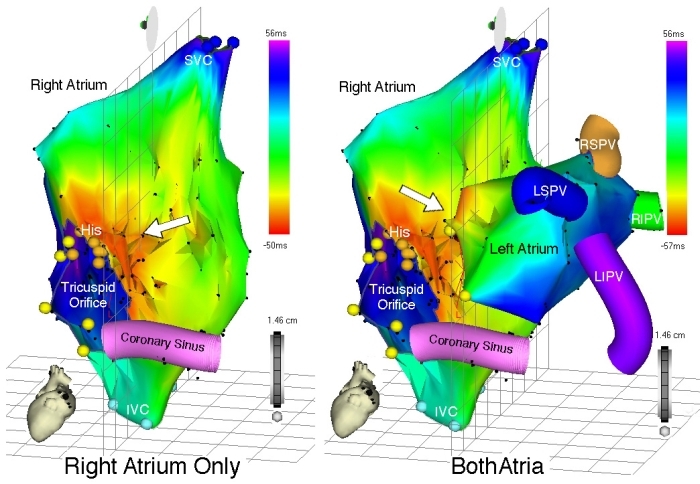
Left panel - Right atrial activation map during focal left AT, left anterior oblique (LAO) view.  Represented anatomic structures include the superior and inferior vena cava (SVC and IVC, respectively), tricuspid orifice, bundle of His (orange points), and CS. Yellow points represent anatomic landmarks from which the tricuspid orifice is reconstructed.  As expected, earliest RA activation occurs over a broad area in the interatrial septum, marked by the red isochrone (arrow) with emanation throughout the RA according to the color-coded time scale.  The right panel shows an activation map of both atria (LAO view) once the LA was accessed, mapped, and the activation data added to the map.  The site of earliest activation is in the medial LA, and the color coded isochrones are adjusted accordingly.  The anatomic locations of the PVs were recorded during LA mapping and displayed.   LS = left superior, LI = left inferior, RS = right superior, RI = right inferior.

**Figure 4:**
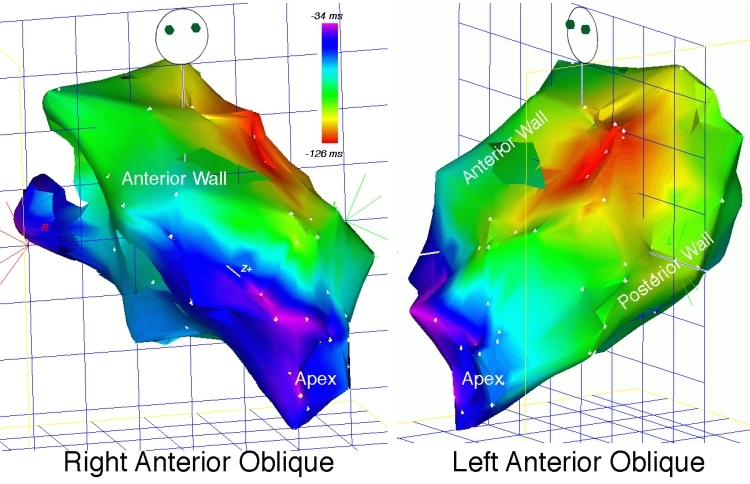
Activation map of focal LV tachycardia, right anterior oblique (RAO) and LAO views.  The red isochrone indicates earliest ventricular activation (and therefore, tachycardia origin) emanating from the anterior LV free wall.

**Figure 5:**
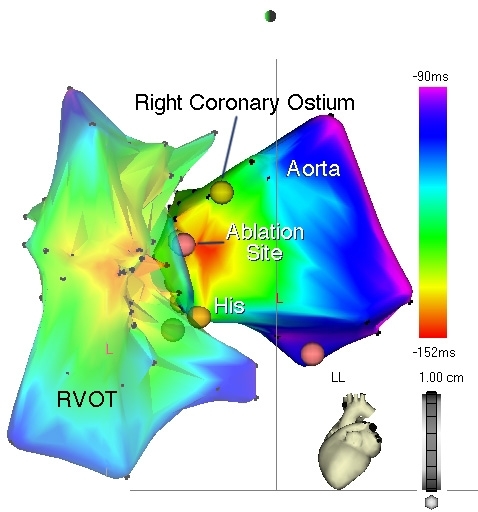
Activation map of the RV outflow tract (RVOT) and aortic sinus in a patient with frequent, symptomatic premature ventricular complexes, left lateral view.  The RVOT was initially mapped and demonstrated early activation (orange isochrone) in its anteroseptal aspect.  Mapping of the aortic sinus, however, revealed even earlier activation as evidenced by the red isochrone, with uniform, centrifugal spread, to the adjacent RVOT.  Tagged landmarks include the His bundle and right coronary artery (RCA) ostium.  The successful ablation site (magenta dot) is approximately 8 mm inferior to the RCA ostium.

**Figure 6:**
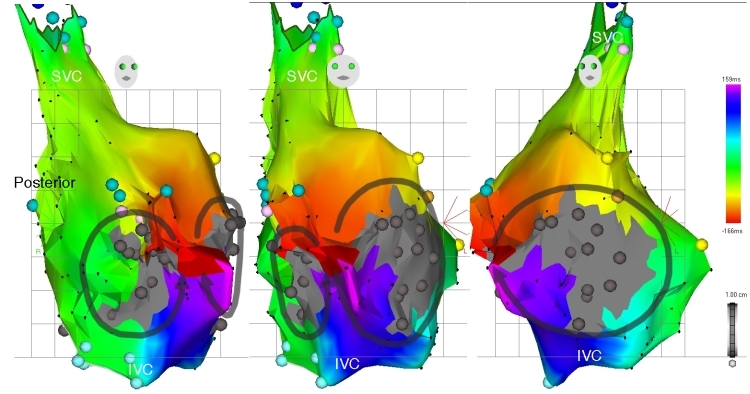
Right atrial activation map of atypical (macroreentrant) atrial flutter, left to right, RAO, anteroposterior (AP), and LAO views. Once again, earliest activation has been defined to record areas of mid-diastolic electrical activity.  This corresponds to a narrow isthmus of atrial tissue (red isochrone) surrounded by large areas of myocardial scar (dense gray regions).  Two separate wavefronts are seen propagating through this isthmus in opposite directions, around two separate islands of scar, according to the color time scale.

**Figure 7:**
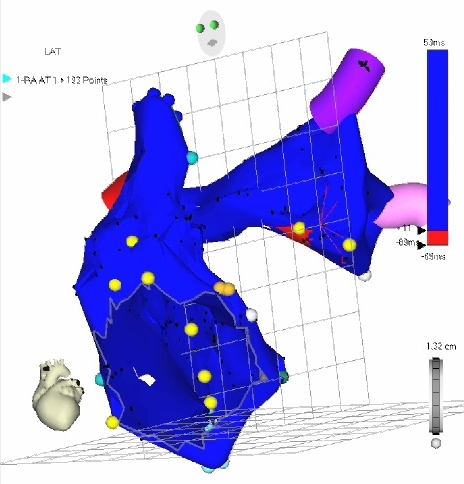

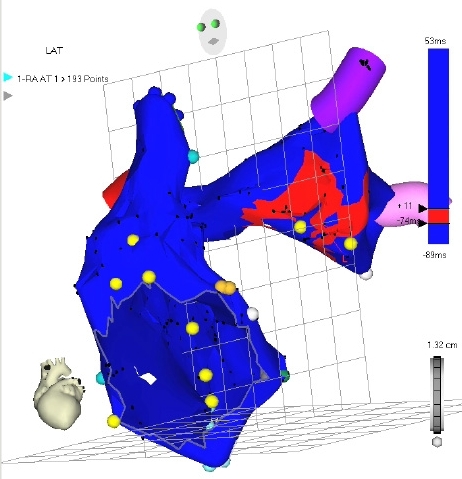

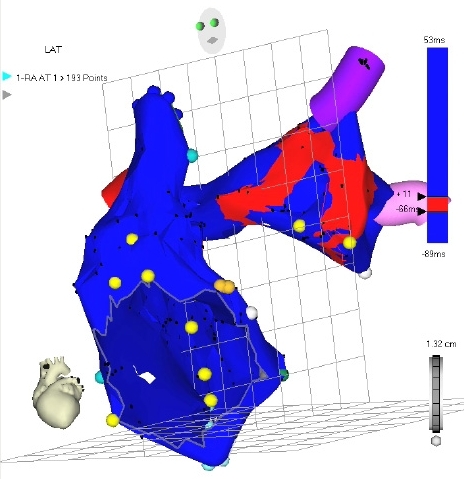

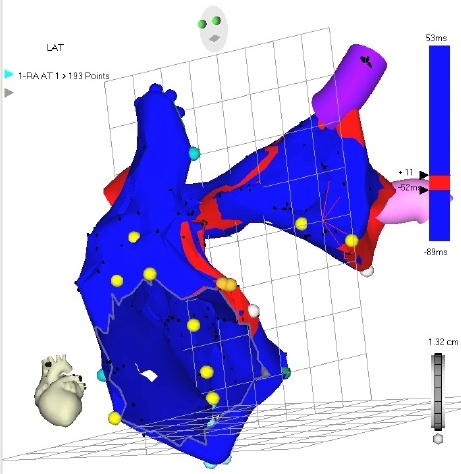

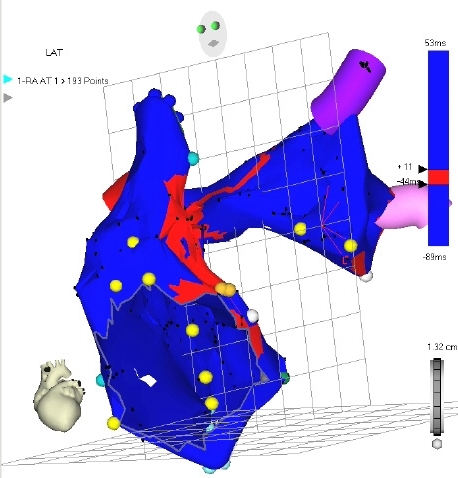

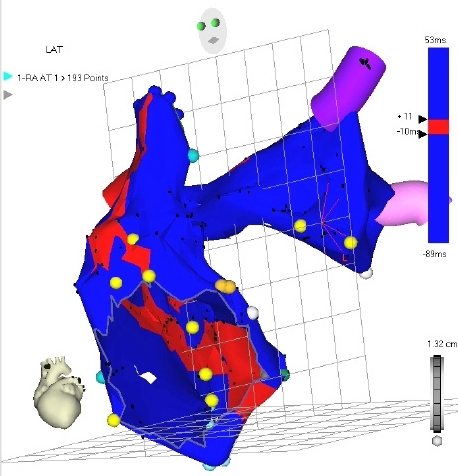

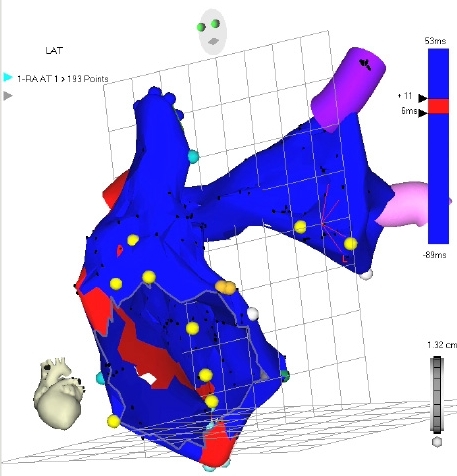

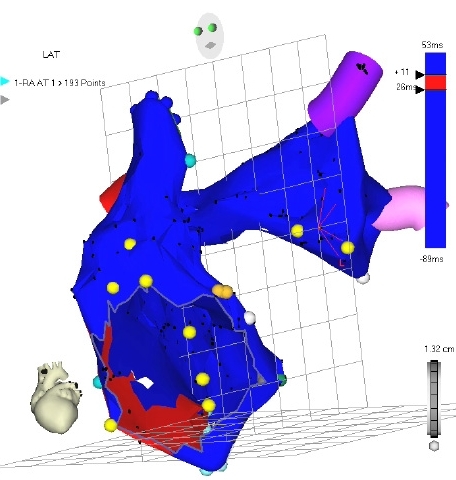
Propagation map of the RA and LA during focal atrial tachycardia, LAO view.  Red coloration indicates atrial activation at different points in the cardiac cycle (proceeding from top to bottom) according to the time scale on the right of each panel.  Atrial activation is seen originating in the lateral LA (top most panel) and emanating in a centrifugal manner to activate the remainder of the LA and entire RA.  The SVC, tricuspid orifice, LSPV, LIPV, and RSPV (red segment visible just posterior to the SVC) have been marked. Cine Version of Figure 7

**Figure 8:**
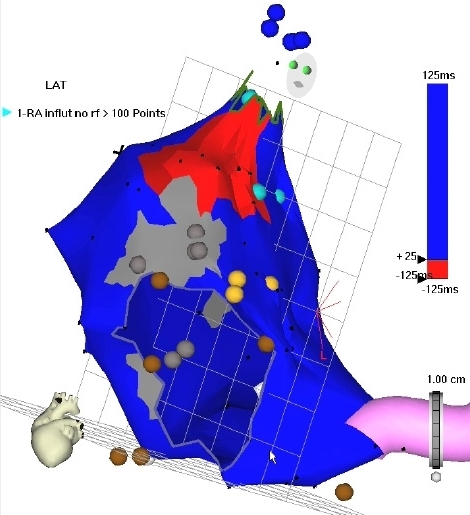

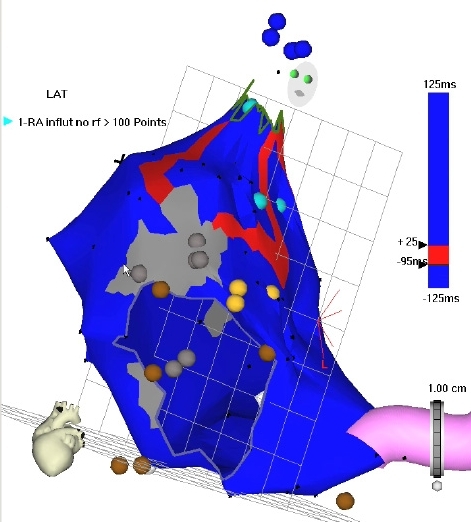

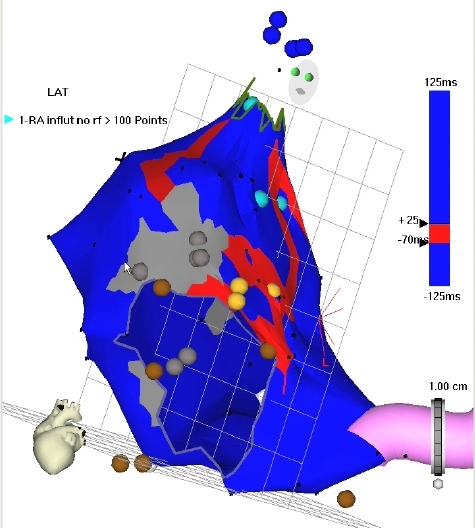

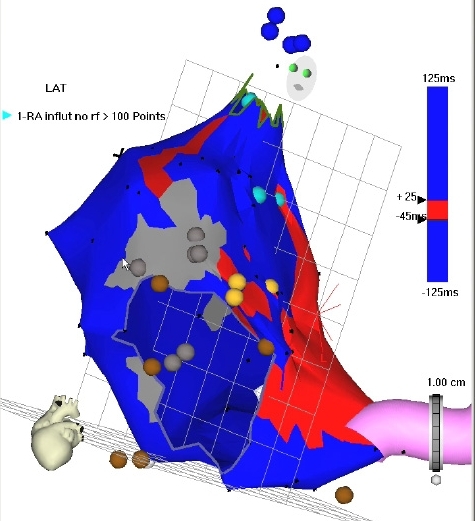

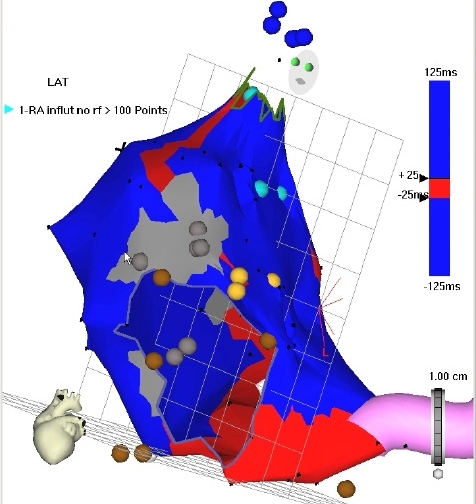

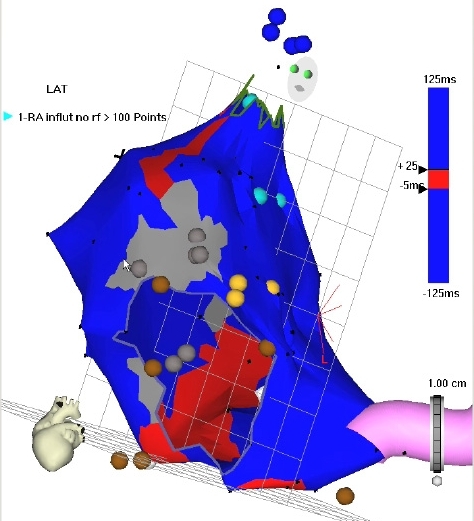

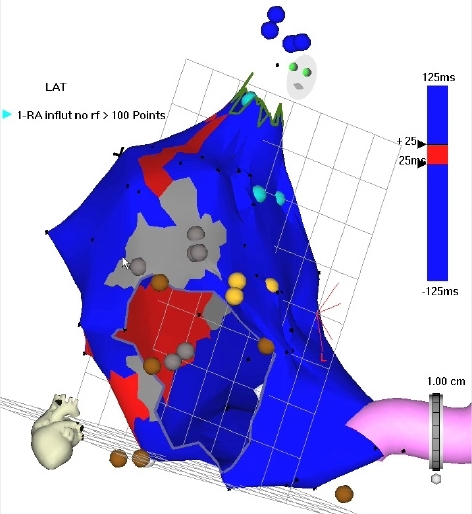

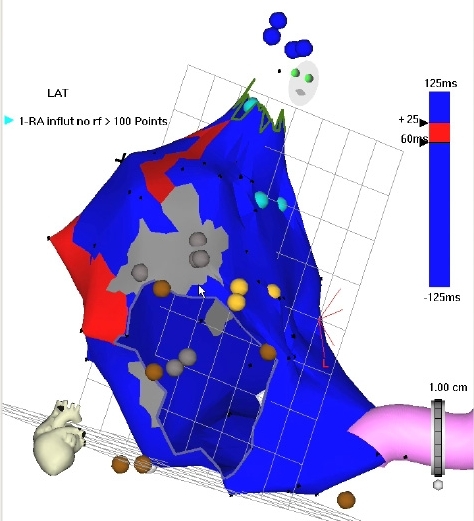

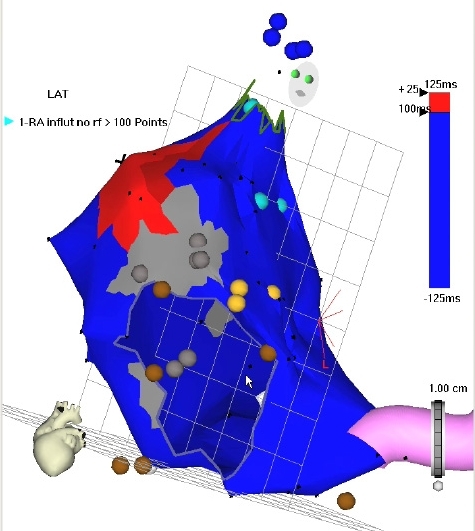
Propagation map of the RA during macroreentrant RA tachycardia, LAO view. Activation is once again demarcated by red coloration, proceeding from left to right. Earliest activation (site of mid-diastolic activity) is seen in the superior RA, adjacent to an area of scar (gray), followed by activation along the medial, inferior, and posterolateral RA, in a circular manner, and according to the time scale.  The SVC, tricuspid orifice, His bundle (light orange points) and CS have been marked. Cine Version of Figure 8

The timing window refers to the range of activation times surrounding reference EGM activation.  Proper timing window definition requires accurate determination of the underlying tachycardia mechanism (focal versus macroreentrant tachycardia); erroneously diagnosed tachycardia mechanism will result in vastly incorrect timing window definition and consequently, creation of an invalid activation map. Once the correct tachycardia mechanism has been diagnosed, the timing window may be defined to include the extremes of cardiac activation preceding and following reference EGM activation. (Figure 2) Propagation maps can also be constructed and played back from data obtained through activation mapping, demonstrating spread of activation throughout a cardiac chamber during arrhythmia.  Figure3,4,5 and 6 are examples of activation maps from focal and macroreentrant mechanisms, and Figures 7 and 8 show propagation maps of focal and reentrant arrhythmias, respectively.

### Anatomic mapping with CARTO

**Figure 9:**
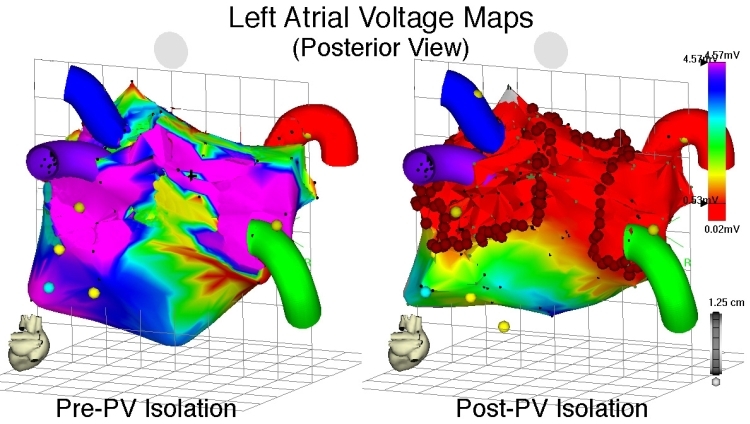
Voltage maps of the LA during sinus rhythm, posteroanterior (PA) view, before (left panel) and after (right panel) wide encircling ablation of the pulmonary veins for atrial fibrillation.  All four PVs have been marked. Local bipolar electrogram voltage of ≤ 0.5 mV has been arbitrarily selected as the threshold for low voltage, as depicted on the voltage scale on the right. The left panel demonstrates relatively normal bipolar electrogram voltage throughout the LA prior to ablation.  The right panel shows large areas of low electrogram voltage (red areas) following ablation, due to conduction block created by encircling ablation lesions (circular red points).

**Figure 10:**
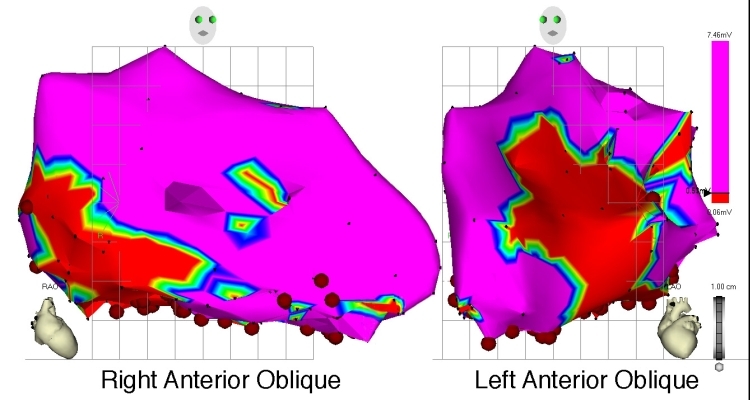
Voltage map of the left ventricle (LV), RAO and LAO view, in an individual with large, healed inferolateral myocardial infarction.  Local bipolar electrogram voltage of ≤ 0.5mV has been arbitrarily selected as the threshold for delineating low electrogram amplitude, as per the voltage scale on the right. The circumscribed area of red coloration represents sites of lowest electrogram amplitude, with the area of next lowest voltage demarcated by yellow, followed by green etc. The area colored in magenta indicates normal electrogram voltage.

**Figure 11:**
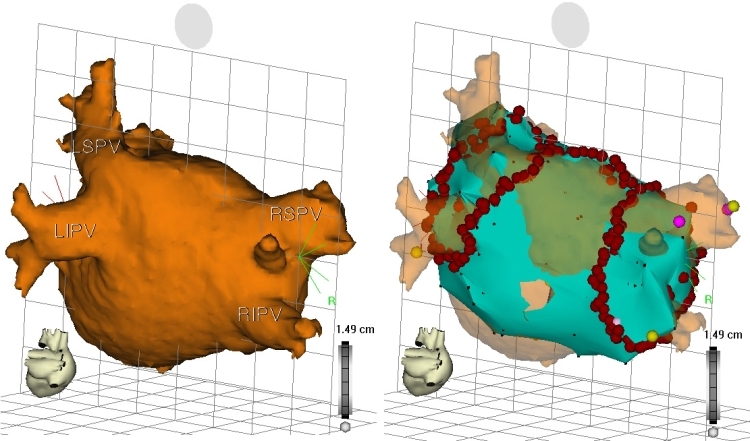
Left panel, reconstructed computed tomography (CT) image of the LA and PVs, PA view.

In addition to facilitating activation mapping, the CARTO system provides location mapping features capable of recording sites of anatomic relevance, areas of low endocardial voltage representing scar, and areas of ablation. Structures such as the bundle of His can be tagged to prevent inadvertent energy delivery resulting in conduction impairment when ablating tachycardias originating in this region. Vessels such as the CS and PVs may also be marked to provide spatial orientation to assist mapping efforts (Figures 3 and 9). Scar mapping can also be achieved by tracing the endocardial surface and recording the amplitude of local potentials. As with endocardial activation, a voltage scale may be arbitrarily chosen to display only the areas of lowest voltage amplitude, to distinguish between areas of scar, dense scar, and relatively normal tissue (Figures 9 and 10). Ablation can be performed to electrically isolate such areas, particularly when only non-sustained or hemodynamically unstable rhythms are induced. The "Remap" feature allows existing chamber geometry to be applied to separate activation maps, when differing arrhythmia morphologies originating from the same chamber are encountered.  In this situation, new activation timing points may be acquired by tracing a previously constructed shell of chamber anatomy. CARTO Merge is another feature that allows for superimposing a previously constructed computed tomography or magnetic resonance imaging and real-time acquired anatomic information through catheter mapping (Figure 11).

Several other EAM systems are presently available for clinical use, each employing differing technology to accomplish the aforementioned tasks.  These systems are briefly reviewed here.

## EnSite NavX® mapping system

**Figure 12:**
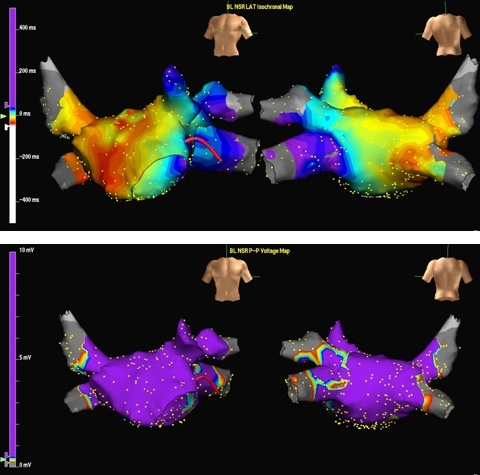

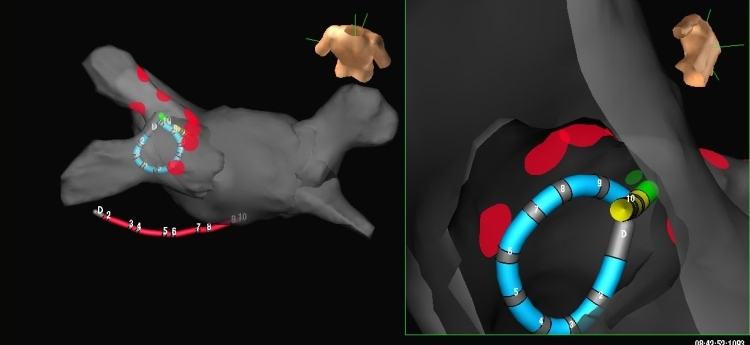
Electroanatomical maps acquired by the Ensite NavX system.  A.  Top.  Activation map of the LA during sinus rhythm, AP and PA views.  Bottom.  Simultaneously-acquired voltage map of the LA, demonstrating low-amplitude EGMs in the PV ostia. B. Posteroanterior view of the LA and PVs.  Left. A decapolar encircling catheter and mapping/ablation catheter have engaged the LSPV ostium.  A decapolar catheter, located within the CS, is also displayed.  Right.  Magnified view of encircling and mapping catheters.

The EnSite NavX® system (Endocardial Solutions, St. Jude Medical, Inc., St. Paul, MN, USA) is capable of displaying 3D positions of multiple catheters.  This is achieved by applying a low-level 5.6 kHz current through orthogonally-located skin patches.  The recorded voltage and impedance at each catheter's electrodes generated from this current allows their distance from each skin patch, and ultimately, their location in space, to be triangulated with the help of a reference electrode.  Three-dimensional images of each catheter can then be displayed.  Respiratory motion artifact can also be eliminated to prevent confounding of actual catheter position [12]. Chamber geometry can be determined thereafter by moving a mapping catheter along the endocardial surface.  (Figure 12)  Human studies have demonstrated feasibility and benefits when the EnSite NavX system is used in conjunction with conventional mapping strategies [19].  In one study, significant reductions in fluoroscopy time, radiation dosage, and procedure time was observed with comparable success in achieving pulmonary isolation when the NavX system was used [12].  Use of NavX was also associated with significantly reduced fluoroscopy time in the treatment of AV node reentry and typical atrial flutter, when compared to standard mapping techniques, without difference in procedure time. Overall, significantly less radiation dose was also seen with NavX [4].  Strengths of the NavX system include the ability to simultaneously display multiple catheter positions in real-time, accurate representation of cardiac anatomy, and its compatibility with any EP catheter. Respiratory artifact is also minimized with current systems. Disadvantages include limited utility against non-sustained arrhythmias, though noncontact mapping can assist in this regard. Inaccurate rendition of complex anatomic structures can also occur unless meticulous contact mapping is performed in such areas [20].

## Noncontact mapping

**Figure 13:**
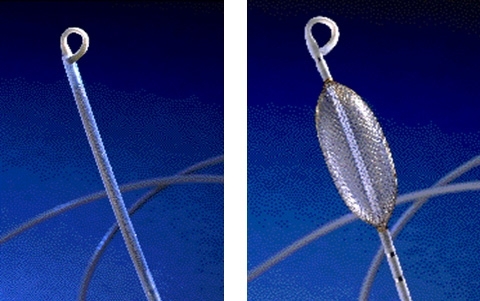
Multielectrode array balloon catheter in its non-deployed (left) and deployed (right) state.

**Figure 14:**
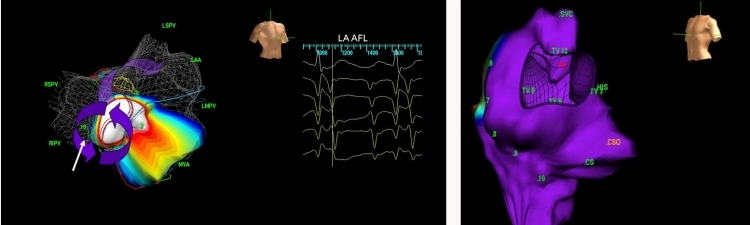
Electroanatomic maps acquired by using a MEA.  Left.  Activation map of macro-reentrant LA flutter. The arrows depict wavefront propagation within the flutter circuit.  Right.  Anatomical reconstruction of the RA.

The noncontact mapping system utilizes a multi-electrode array (MEA) catheter (Ensite, Endocardial Solutions Inc., St. Paul, MN, USA) to simultaneously record multiple areas of endocardial activation [21]. The MEA is an inflatable balloon with 64 electrodes on its surface.  (Figure 13)  Hence, relatively high-density mapping can be performed from even a single beat of tachycardia. Three-dimensional (3D) localization of the MEA surface electrodes is achieved by applying a low-level 5.6 kHz current between an electrode on the distal end of the MEA catheter and two ring electrodes along its shaft, proximal and distal to the MEA itself.  Similarly, a separate mapping catheter's position can be determined by delivering current between its electrode and these two ring electrodes. Chamber geometry can be reconstructed by manipulating this mapping catheter within the chamber of interest and the corresponding electrical isopotentials can be "plotted" on this geometric representation of the endocardial surface [21,22].  (Figure 14) In one animal study, the EnSite mapping system demonstrated accurate representation of local electrograms obtained from the MEA (both timing and waveform correlation) when compared to recordings taken from a standard mapping catheter. Accuracy of activation mapping was also assessed in this study by measuring the mean distance between the center and edge of ablation lesions from mapping of a target LV pacing site (4.0 ± 3.2 mm and 1.2 ± 3.2 mm, respectively). In vitro testing showed the EnSite system to have excellent locator precision and accuracy at distances <50 mm from the MEA, though these parameters were less impressive at distances >50 mm.(21)  In one human LV mapping study, points were separated according to their location on or away from the MEA equatorial plane.  Electrogram timing and morphology from equatorial points obtained from the noncontact MEA correlated well with contact electrograms, though worsened with greater distance from the MEA: timing correlation significantly worsened at a threshold distance of >34 mm from the MEA center; a threshold distance for worsening electrogram morphology was not as clear.  For non-equatorial points, correlation of electrogram timing and morphology was comparable to that observed with equatorial points; a threshold distance where significant worsening of these parameters occurred was not observed [22]. Advantages of the EnSite mapping system include acquisition of multiple endocardial electrograms from a single beat, rendering it quite useful in mapping atrial and ventricular ectopic beats, non-sustained arrhythmias, and rhythm disturbances that are poorly tolerated hemodynamically. Any mapping/ablation catheter is also compatible with this system. Disadvantages include inaccuracy of electrogram timing and morphology at greater distances from the MEA, difficulty in positioning the MEA balloon, and inaccuracy in reconstructing certain features of chamber geometry [20].

## Real-Time Position Management System

The Real-Time Position Management (RPM) System (Cardiac Pathways, Sunnyvale, CA, USA) employs ultrasound ranging to localize reference and mapping/ablation catheter positions [23]. Two reference catheters, one typically situated in the RA, CS, or RV, and the mapping/ablation catheter each contain an ultrasound transducer along their shaft. A separate ultrasound transmitting and receiving device emits continuous ultrasound energy at 558.5 kHz. This energy is received by the transducers housed within the reference and ablation catheters; the time required to receive this signal by each transducer is converted to a distance (by utilizing a constant velocity of sound waves in blood) and the position of each catheter is thereby determined. Accuracy of this system has been verified in basic validation studies [23].  Its feasibility and reliability in mapping atrial flutter, ventricular tachycardia, and accessory pathway-mediated tachycardia in humans have also been established [23].  Specific advantages of the RPM system include continuous real-time location of ablation and reference catheters, the ability to demonstrate the degree of catheter deflection, and the capability of repositioning reference catheters to their original location should they become dislodged, as these locations can be stored.  Disadvantages of this system include potential limitations in recording anatomic features between separate cardiac structures (such as the left atrium (LA) and pulmonary veins [PVs]) that may lead to distortion of cardiac geometry, and the need to use specific reference and ablation catheters equipped with ultrasound transducers [20,23].

Table 1 summarizes currently available EAM systems, and each system's strengths and weaknesses.

**Figure 15:**
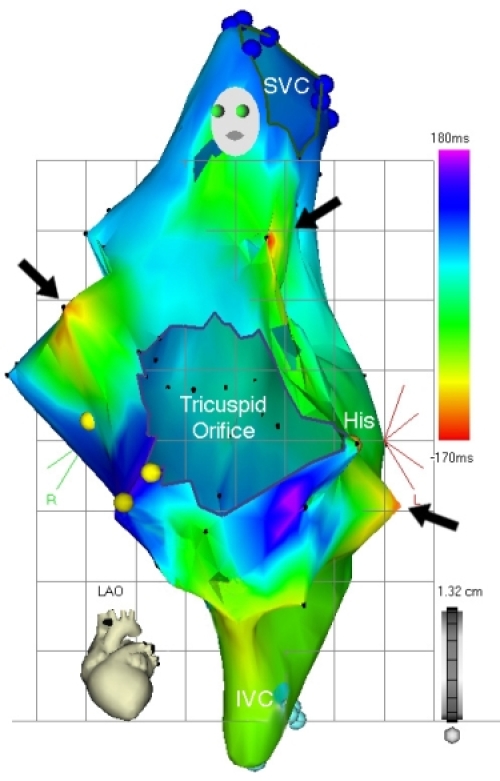

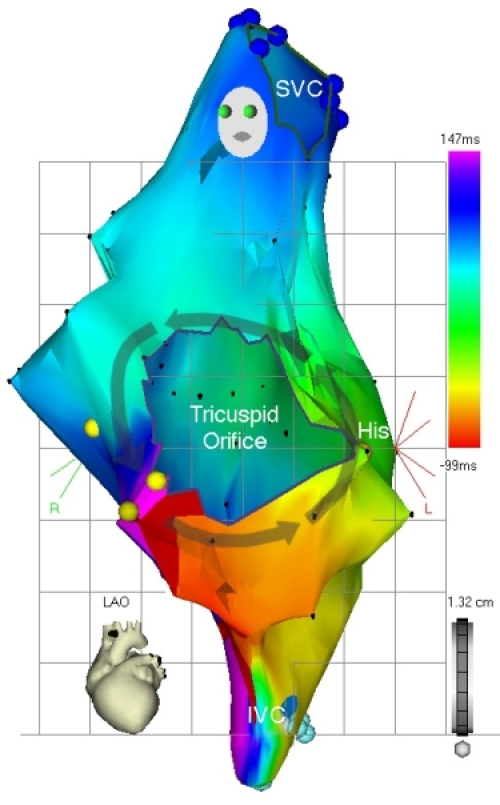
Upper panel.  Right atrial activation map of right atrial flutter (LAO view) obtained from using erroneous tachycardia cycle length of 350 ms.  Several different areas of red isochrones (area) indicating early activation are seen, yielding a confusing activation map. Lower panel. Once the proper tachycardia cycle length (250 ms) has been applied, typical, counterclockwise, cavo-tricuspid isthmus dependent flutter is evident.  "Earliest" activation has been defined to record areas of mid-diastolic electrical activity, and is represented by the red isochrone, anatomically corresponding to the cavo-tricuspid isthmus.  Wavefront propagation in a counterclockwise direction around the tricuspid annulus is seen according to the color time scale.

**Figure 16:**
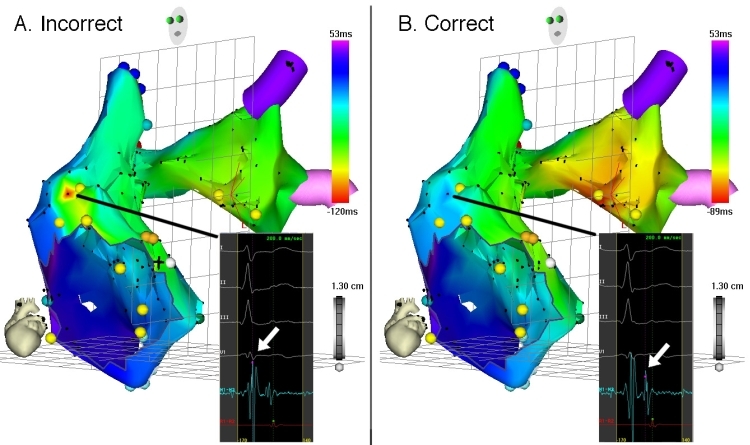
Left panel, activation map of the RA and LA of what was ultimately diagnosed as left AT.  The area of earliest activation (red isochrone) is observed to be in the anterolateral RA, however.  Inspection of the activation window (inset) corresponding to this seemingly early-activated point shows it was incorrectly recorded, using the ventricular, rather than the atrial EGM (arrow). Note the confusing features of this map: a very circumscribed area of early activation bordered by a large area of late activation (dark blue isochrone), accompanied by an adjacent area of intermediate-early activation (green and aqua isochrones) in the RA, and nearly uniform intermediate-early activation throughout the LA. Right panel, the erroneous activation map is rectified by correctly using the atrial EGM (arrow, inset) at aforementioned point.  The resulting activation map correctly shows earliest activation in the LA with uniform spread throughout both atria.  Ablation in the area of the red isochrone successfully eliminated AT.

While EAM systems may greatly facilitate mapping efforts, reduce procedure time and fluoroscopic exposure, they must never replace fundamental principles of arrhythmia diagnosis and sound decision making; at most, such systems must be used as an adjunctive tool when approaching a target arrhythmia.  Wholly relying on data from an incorrectly acquired map may lead to an erroneously diagnosed arrhythmia mechanism, inaccurate site of tachycardia origination, or creation of a confusing, unhelpful activation map. Such errors may counteract any possible benefit offered by EAM, and may ultimately prolong procedure time and increase fluoroscopic exposure. (Figures 15 and 16)  It must also be remembered that while EAM system use may seem ubiquitous nowadays, procedural success rates prior to their widespread application are comparable to what is presently observed [1-7].

## Conclusion

In summary, several EAM systems are available to facilitate cardiac arrhythmia mapping and ablation efforts, each distinguished by differing technology, and each with its own merits and weaknesses.  The decision to use a particular system depends on the data hoped to be gained by EAM (activation mapping, substrate mapping, cardiac geometry), the anticipated arrhythmia to be targeted, the compatibility of the system with adjunctive tools (i.e. diagnostic and ablation catheters), and perhaps most importantly, the user's familiarity with the chosen EAM system.  While these systems provide a wealth of data and can substantially facilitate mapping efforts, reduce procedure time and fluoroscopic exposure, they cannot replace careful interpretation of data and strict adherence to electrophysiologic principles.

## Supplementary Material

Cine Version of Figure 7

Cine Version of Figure 8

## Figures and Tables

**Table 1:**